# Prevaccination Glucose Time in Range Correlates With Antibody Response to SARS-CoV-2 Vaccine in Type 1 Diabetes

**DOI:** 10.1210/clinem/dgad001

**Published:** 2023-01-06

**Authors:** Ghadeer Alhamar, Silvia Briganti, Daria Maggi, Viola Viola, Malak Faraj, Carla Zannella, Massimiliano Galdiero, Gianluigi Franci, Clorinda Fusco, Camilla Isgrò, Giulia Leanza, Ilaria Malandrucco, Andrea Spinelli, Flavia Tramontana, Domenico Iaria, Rachele Tortoriello, Silvia Pieralice, Milena Rosati, Giuseppe Matarese, Paolo Pozzilli, Mario Galgani, Rocky Strollo

**Affiliations:** Department of Medicine, Endocrinology & Diabetes Unit, Università Campus Bio-Medico di Roma, 00128 Rome, Italy; Dasman Diabetes Institute, 15462 Kuwait City, Kuwait; Department of Medicine, Endocrinology & Diabetes Unit, Università Campus Bio-Medico di Roma, 00128 Rome, Italy; Department of Medicine, Endocrinology & Diabetes Unit, Università Campus Bio-Medico di Roma, 00128 Rome, Italy; Department of Medicine, Endocrinology & Diabetes Unit, Università Campus Bio-Medico di Roma, 00128 Rome, Italy; Department of Medicine, Endocrinology & Diabetes Unit, Università Campus Bio-Medico di Roma, 00128 Rome, Italy; Dipartimento di Medicina Sperimentale, Università degli Studi della Campania “Luigi Vanvitelli,” 80138 Naples, Italy; Dipartimento di Medicina Sperimentale, Università degli Studi della Campania “Luigi Vanvitelli,” 80138 Naples, Italy; Dipartimento di Medicina Chirurgia ed Odontoiatria “Scuola Medica Salernitana,” Università degli Studi di Salerno, 84081 Baronissi, Italy; Istituto per l’Endocrinologia e l’Oncologia Sperimentale “G. Salvatore,” Consiglio Nazionale delle Ricerche, 80131 Naples, Italy; Unità di Neuroimmunologia, Istituto di Ricovero e Cura a Carattere Scientifico (IRCCS), Fondazione Santa Lucia, 00179 Rome, Italy; Department of Medicine, Università Campus Bio-Medico di Roma, 00128 Rome, Italy; Department of Basic Medical Sciences, Neurosciences and Sense Organs, University of Bari “Aldo Moro,” 70121 Bari, Italy; Department of Medicine, Endocrinology & Diabetes Unit, Università Campus Bio-Medico di Roma, 00128 Rome, Italy; Unità Operativa Semplice Dipartimentale Endocrinologia e Malattie Metaboliche, Azienda Sanitaria Locale (ASL) Frosinone, 03100 Frosinone, Italy; Department of Science and Technology for Humans and the Environment, Università Campus Bio-Medico di Roma, 00128 Rome, Italy; Department of Medicine, Endocrinology & Diabetes Unit, Università Campus Bio-Medico di Roma, 00128 Rome, Italy; Department of Medicine, Endocrinology & Diabetes Unit, Università Campus Bio-Medico di Roma, 00128 Rome, Italy; Department of Medicine, Endocrinology & Diabetes Unit, Università Campus Bio-Medico di Roma, 00128 Rome, Italy; Department of Medicine, Endocrinology & Diabetes Unit, Università Campus Bio-Medico di Roma, 00128 Rome, Italy; Department of Medicine, Endocrinology & Diabetes Unit, Università Campus Bio-Medico di Roma, 00128 Rome, Italy; Istituto per l’Endocrinologia e l’Oncologia Sperimentale “G. Salvatore,” Consiglio Nazionale delle Ricerche, 80131 Naples, Italy; Dipartimento di Medicina Molecolare e Biotecnologie Mediche, Università degli Studi di Napoli “Federico II,” 80131 Naples, Italy; Department of Medicine, Endocrinology & Diabetes Unit, Università Campus Bio-Medico di Roma, 00128 Rome, Italy; Istituto per l’Endocrinologia e l’Oncologia Sperimentale “G. Salvatore,” Consiglio Nazionale delle Ricerche, 80131 Naples, Italy; Dipartimento di Medicina Molecolare e Biotecnologie Mediche, Università degli Studi di Napoli “Federico II,” 80131 Naples, Italy; Department of Science and Technology for Humans and the Environment, Università Campus Bio-Medico di Roma, 00128 Rome, Italy

**Keywords:** SARS-CoV2, mRNA vaccine BNT162b2, type 1 diabetes, glucose control, continuous glucose monitoring, neutralizing antibodies

## Abstract

**Context:**

Poor glucose control has been associated with increased mortality in COVID-19 patients with type 1 diabetes (T1D).

**Objective:**

This work aimed to assess the effect of prevaccination glucose control on antibody response to the SARS-CoV-2 vaccine BNT162b2 in T1D.

**Methods:**

We studied 26 patients with T1D scheduled to receive 2 doses, 21 days apart, of BNT162b2, followed prospectively for 6 months with regular evaluation of SARS-CoV-2 antibodies and glucose control. Immunoglobulin G (IgG) to spike glycoprotein were assessed by enzyme-linked immunosorbent assay, and serum neutralization by a live SARS-CoV-2 assay (Vero E6 cells system). Glycated hemoglobin A_1c_ (HbA_1c_) and continuous glucose monitoring (CGM), including time in range (TIR) and above range (TAR), were collected. The primary exposure and outcome measures were prevaccination glucose control, and antibody response after vaccination, respectively.

**Results:**

Prevaccination HbA_1c_ was unrelated to postvaccine spike IgG (*r* = −0.33; *P* = .14). Of note, the CGM profile collected during the 2 weeks preceding BNT162b2 administration correlated with postvaccine IgG response (TIR: *r* = 0.75; *P* = .02; TAR: *r* = −0.81; *P* = .008). Patients meeting the recommended prevaccination glucose targets of TIR (≥ 70%) and TAR (≤ 25%) developed stronger neutralizing antibody titers (*P* < .0001 and *P* = .008, respectively), regardless of HbA_1c_. Glucose control along the study time frame was also associated with IgG response during follow-up (TIR: *r* = 0.93; *P* < .0001; TAR: *r* = −0.84; *P* < .0001).

**Conclusion:**

In T1D, glucose profile during the 2 weeks preceding vaccination is associated with stronger spike antibody binding and neutralization, highlighting a role for well-controlled blood glucose in vaccination efficacy.

Poor glucose control has been associated with increased mortality in COVID-19 patients with type 1 diabetes (T1D) (1). For example, a population-based cohort study of 264 390 people with T1D showed that COVID-19–related mortality was significantly higher in patients with a glycated hemoglobin A_1c_ (HbA_1c_) greater than or equal to 10.0% compared to people with an HbA_1c_ of 6.5 to 7.0% (hazard ratio 2.23; 95% CI, 1.50-3.30) (1). However, whether glucose control may also affect immunogenicity to SARS-CoV-2 vaccines is not clear. Thus, the aim of this study was to assess the effect of prevaccination glucose control, measured by HbA_1c_ and continuous glucose monitoring (CGM), on antibody response induced by SARS-CoV-2 vaccination in patients with T1D. We hypothesized that lower HbA_1c_ or a better CGM profile during the prevaccination period would lead to a greater antibody response to the vaccine. Compared to HbA_1c_, CGM provides reliable assessment of glucose along shorter periods of time (2, 3), thus being ideal for studying the effect of glucose recorded during time frames crucial for mounting the immune response to SARS-CoV-2 vaccine (4).

## Materials and Methods

### Research Design and Participants

This was a single-center, 6-month cohort study of T1D patients scheduled to receive 2 doses (30 μg messenger RNA [mRNA] per dose), 21 days apart, of the SARS-CoV-2 mRNA vaccine BNT162b2, carried out between April 2021 and November 2021. Inclusion criteria were patients aged 18 years or older with T1D. Exclusion criteria were previous known SARS-CoV-2 infection, pregnancy or breastfeeding, end-stage renal failure, neoplastic diseases, and immunosuppressive therapies. Immunoglobulin G (IgG) antibodies to spike glycoprotein were assessed at 5 time points: within 3 days before the first vaccine dose (baseline, T0); 21 days from baseline (just before the second dose, T1); 35 days from baseline (2 weeks after the second dose, T2); and 90 (T3) and 180 (T4) days from baseline.

### Ethical Approval

All clinical investigations were conducted according to the principles expressed in the Declaration of Helsinki. This study was approved by the Comitato Etico Università Campus Bio-Medico di Roma Ethical Committee (No. Prot. PAR33-21; approval date April 13, 2021).

### Detection of Spike Antibodies

Antibody binding to spike protein was evaluated by a standard enzyme-linked immunosorbent assay (ELISA) protocol (5), adapted from Amanat et al (6). Briefly, 96-well Nunc ELISA plates were coated with 50 μL of 2 μg/mL of SARS-CoV-2 spike protein (10549-CV-MTO, R&D Systems) and incubated overnight at 4 °C. After blocking for 1 hour with 3% skimmed milk in 0.1% Tween–phosphate-buffered saline (PBS), 100 μL of 1:1280-diluted serum samples in 1% skimmed milk Tween-PBS were added to each well, followed by 1-hour incubation at room temperature. The plates were then incubated with 100 μL of rabbit anti-human IgG–horseradish peroxidase (HRP) (Millipore catalog No. AP101P, RRID:AB_92409) at 1:3000 dilution for 1 hour. Then, 100 μL of tetramethylbenzidine substrate (Sigma) in 100 mmol/L sodium acetate (pH 6.0) were added to each well and the reaction stopped with 20% sulfuric acid. The optical density (OD) was read at 450 nm using a GENios plate reader and Magellan software (Tecan). Between steps the plates were washed 4 times with 0.05% Tween-PBS. Two known native β-cell autoantigens, insulin and GAD65, were used as positive control antigens, and specific autoantibodies were determined as previously described (7, 8). Each assay included 4 known positive reference control samples (2 high binders, 1 medium-high binder, 1 medium-low binder) and 1 negative reference control sample. Intra-assay coefficient of variation of duplicates was less than 8%; interassay coefficient of variation was less than 12%. In our laboratory, the assay achieved 88% sensitivity and 99% specificity for identifications of COVID-19 among 115 individuals (56 COVID-19 patients).

### SARS-CoV-2 Microneutralization Assay

Serum neutralization potency was assessed by a live SARS-CoV-2 assay using the Vero E6 cells system as previously described (9, 10). Serum samples were stored at −20 °C, diluted (1:10; 1:40; 1:160; 1:640) in triplicate and mixed with 100 TCID50 of SARS-CoV-2 (clinical isolate, strain VR PV10734, kindly donated by the Lazzaro Spallanzani Hospital of Rome, Italy). Then each mixture serum/virus was transferred to 96 wells in the presence of 5 × 10^5^/mL Vero E6 (CRL-1586, ATCC) cells. Monolayers were incubated at 37 °C until the evaluation of cytopathic effect via microscope. Then, cells were stained with crystal violet solution. Neutralization titers of serum samples were calculated by the highest serum dilution protecting 20% (IC20), 50% (IC50), and 90% (IC90) of the infected wells (6, 7). All procedures were carried out in a Biosafety Level 3 (BSL-3) laboratory.

### Glucose Control

Glucose control was assessed by HbA_1c_ (at baseline) and CGM by FreeStyle Libre or Dexcom G6 (at each time point), defined according to the international consensus on time in range (TIR) (2). CGM measurement of the 10 to 14 days before data collection were available for 13 patients with T1D and included percentage of readings within the target glucose range of 70 to 180 mg/dL (TIR), time below (TBR), and time above target glucose range (TAR).

### Statistical Analyses

The primary exposure and outcome measures were prevaccination glucose control assessed by HbA_1c_, and antibody response after vaccination, respectively. The secondary exposure measure was CGM-derived TIR. IgG area under the curve (AUC) was calculated using the trapezoidal method, and then divided by the time period of the test. Longitudinal changes of IgG antibodies were assessed by Friedman test. Correlation analyses were assessed by Spearman test. Using a statistical significance level of .05% and 80% power, the minimum sample size required was 25 participants. This was based on an expected mean ± SD of the difference between peak IgG (T2) and the lowest IgG level at T4 of 0.3 ± 0.5 OD. The sample size was powered to detect large effect size in correlation analyses (eg, *r* > 0.6).

## Results

**Figure 1.**
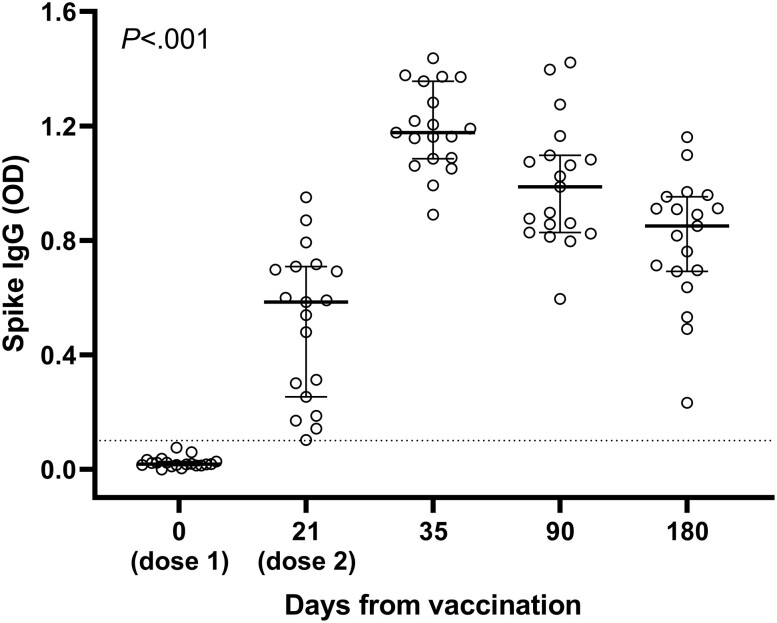
Longitudinal changes in spike immunoglobulin G (IgG) binding. IgG antibody response to spike glycoprotein in patients with type 1 diabetes. Longitudinal IgG response to spike reached a peak response at day 35 (T2), followed by a progressive decline across later time points (*P* < .001).

We enrolled 26 patients with T1D (53.8% male, median age 40 years [interquartile range, 27-48.75 years], disease duration 22 years [13-27.5 years], body mass index [BMI] 24.7 [21.55-27.65], HbA_1c_ 7.4% [6.9-7.7], TIR 61.5% [55.5-71.7], TAR 28.0% [21.0-41.0], TBR 3.0% [2.0-7.5]), treated with continuous subcutaneous insulin infusion (34.6%) or multiple daily injections of insulin (65.4%) according to a basal bolus regimen (Table 1). All patients tested negative to anti-SARS-CoV-2 spike IgG at baseline. After vaccination, the IgG significantly increased and reached a peak at T2, followed by a progressive decline across later time points (*P* < .001; Fig. 1).

**Table 1.** Baseline clinical and glucose control features of the study population

	Type 1 diabetes (n = 26)
Age, y	40 (27-48.75)
Male sex	14 (53.8%)
BMI	24.7 (21.55-27.65)
HbA_1c_, %	7.4% (6.9-7.7)
Microvascular complications, n (%)	6 (23.1%)
Macrovascular complications, n (%)	0 (0.0%)
Additional autoimmune disease, n (%)^*a*^	10 (38.5%)
MDI, n (%)	17 (65.4%)
Pump users, n (%)	9 (34.6%)
CGM users, n (%)	13 (50.0%)
TIR, %	61.5% (55.5-71.7)
TAR, %	28.0% (21.0-41.0)
TBR, %	3.0% (2.0-7.5)

Abbreviations: BMI, body mass index; CGM, continuous glucose monitoring; HbA_1c_, glycated hemoglobin A_1c_; MDI, multiple daily insulin injections; TAR, time above range; TBR, time below range; TIR, time in range.

Additional autoimmune diseases include autoimmune thyroid disease, celiac disease, and/or vitiligo.

**Figure 2.**
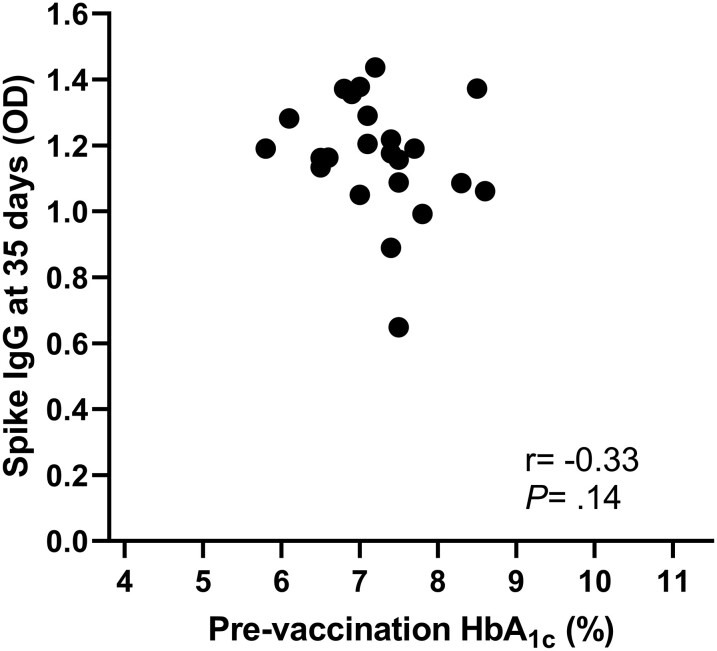
Correlation between prevaccination glycated hemoglobin A_1c_ (HbA_1c_) and spike immunoglobulin G (IgG) antibodies following vaccination. Prevaccination HbA_1c_ was unrelated to spike IgG at T2 (*r* = −0.33; *P* = .14).

**Figure 3.**
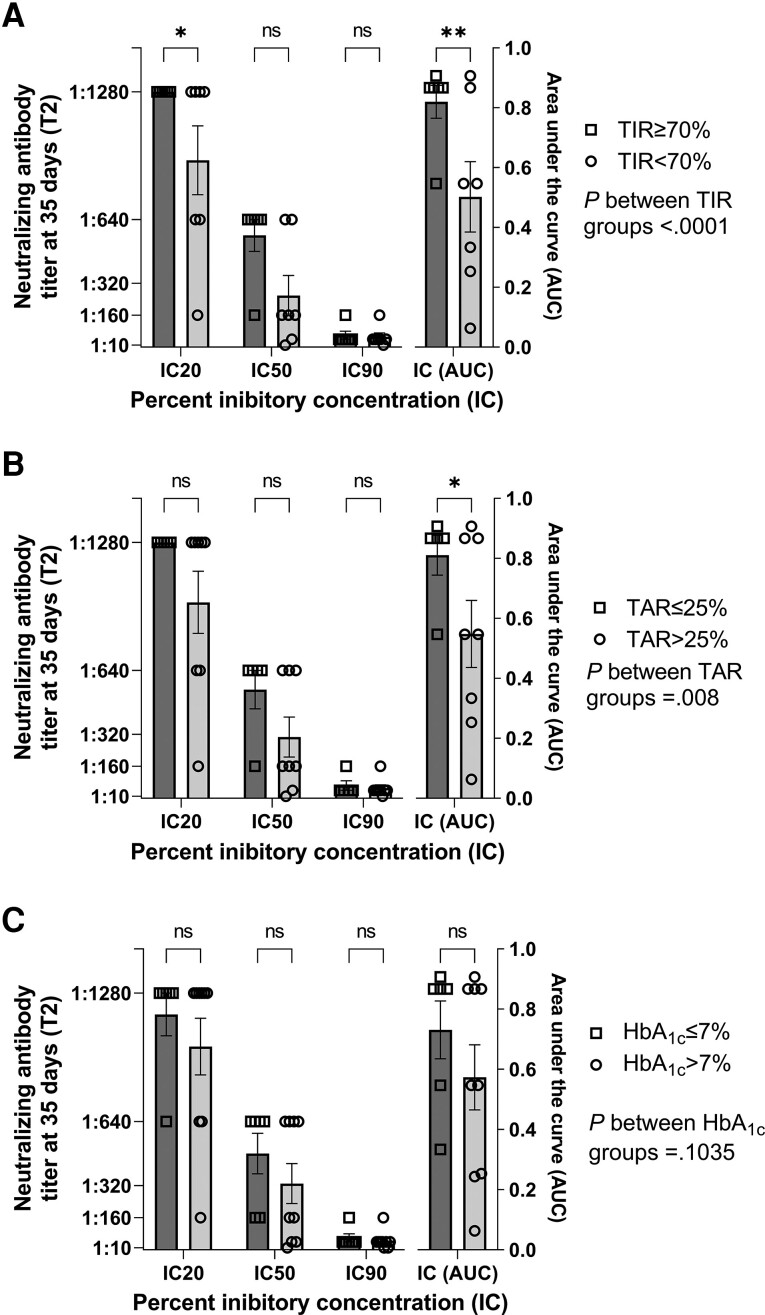
Association between glucose control and neutralization titer in type 1 diabetes. When divided according to recommended blood glucose targets of time in range (TIR, cutoff 70%) and time above range (TAR, cutoff 25%), patients meeting target TIR (≥ 70%) and TAR (< 25%) developed stronger neutralizing antibody titers at 35 days after vaccination compared to patients with a worse TIR and TAR (A, *P* < .0001 and B, *P* = .008, respectively), regardless of C, glycated hemoglobin A_1c_ (HbA_1c_) (n = 13). IC, percentage inhibitory concentration; ns, not significant. *P less than .05; **P less than .01.

**Figure 4.**
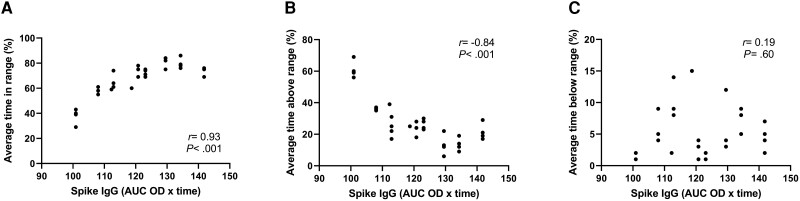
Correlation between time-dependent glucose profile and spike antibody binding after vaccine. Time-dependent mean of time in range (TIR, A) and time above range (TAR, B), but not time below range (TBR, C), collected along 6 months’ follow-up strongly correlated with the area under the curve (AUC) of immunoglobulin G (IgG) response across the 5 time points. IgG antibody binding is expressed as optical density (OD) measured at 450 nm × time.

**Figure 5.**
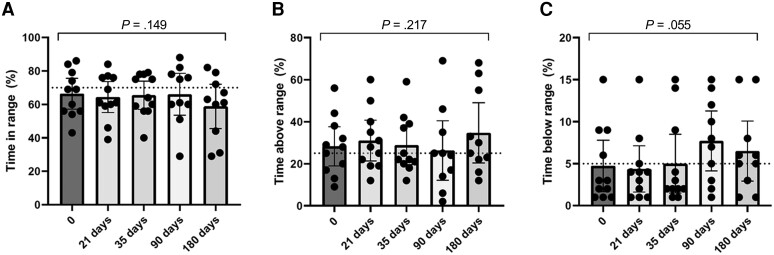
Changes in continuous glucose monitoring in the cohort of patients with type 1 diabetes. Glucose control assessed by continuous glucose monitoring did not change significantly after vaccine administration neither compared to baseline nor between the study time frames (TIR: *P* = .149; TAR: *P* = .217; TBR: *P* = .055).

Prevaccination HbA_1c_ was unrelated to spike antibody response (correlation between HbA_1c_ and peak IgG at T2: *r* = −0.33; *P* = .14; Fig. 2). Of note, in the CGM subgroup of patients, both baseline TIR and TAR strongly correlated with the IgG-AUC over the 6-month study time frame (TIR: *r* = 0.75; *P* = .02; TAR: *r* = −0.81; *P* = .008). The relationship was driven by the peak IgG, which strongly correlated with baseline TIR (n = 13, *r* = 0.70; *P* = .0089) and TAR (n = 13, *r* = −0.70; *P* = .0102), respectively. Peak IgG (T2) also correlated with glucose collected at the same time point, which refers to the glucose control of the 14 days preceding the second vaccine dose (Table 2). Furthermore, patients meeting the recommended blood glucose targets of TIR (≥ 70%) and TAR (≤ 25%) at baseline developed stronger neutralizing antibody titers (*P* < .0001; Fig. 3A; and *P* = .008, Fig 3B; respectively), regardless of HbA_1c_ (Fig. 3C), indicating that the longer the time spent within target glucose levels at baseline, the greater the neutralization potency induced by the SARS-CoV-2 vaccine. Glucose control along the study time frame was also associated with IgG response as shown by the correlation between time-dependent mean of TIR and TAR during follow-up and IgG-AUC (TIR: *r* = 0.93; *P* < .0001; TAR: *r* = −0.84; *P* < .0001; Fig. 4). TBR was unrelated to either peak-IgG or IgG-AUC (−0.04 < *r* < −0.018; *P* > .90), or serum neutralization (*P* = .87). When divided according to recommended blood glucose targets of TIR (cutoff 70%), TAR (cutoff 25%), and HbA_1c_ (cutoff 7%), patients meeting the target TIR (≥ 70%) and TAR (< 25%) at baseline developed stronger IgG responses over time compared to patients with worse TIR and TAR (*P* < .001 and *P* < .05, respectively), regardless of HbA_1c_. A similar effect was found when patients were categorized according to the time-dependent mean of TIR and TAR. The positive correlation between TIR and antibody response was maintained also when the more stringent TIR of 70 to 140 mg/dL was considered (correlation between prevaccination TIR and peak IgG: *r* = 0.69; *P* = .05; correlation between time-dependent mean of TIR and IgG-AUC: *r* = 0.88; *P* = .03). Glucose profile assessed by CGM did not change significantly after vaccine administration compared to baseline or between the study time frames (TIR: *P* = .149; TAR: *P* = .217; TBR: *P* = .055; Fig. 5).

**Table 2.** Correlation between continuous glucose monitoring and antibody levels

	TIR	TAR	TBR
T1, 21 d	*r* = 0.1337	*r* = −0.2606	*r* = 0.4039
	*P* = .7133	*P* = .4697	*P* = .2484
T2, 35 d	***r* = 0.7431**	***r* = −0.7215**	*r* = 0.4908
	***P* = .0111**	***P* = .0148**	*P* = .1268
T3, 90 d	*r* = 0.5471	*r* = −0.5593	*r* = −0.006
	*P* = .1061	*P* = .0951	*P* > .999
T4, 180 d	*r* = 0.4524	*r* = −0.6190	*r* = 0.1091
	*P* = .2675	*P* = .1150	*P* = .8302

The table shows the correlation of continuous glucose monitoring measurements recorded during the 14 days preceding serum collection for antibody assessment. Antibody levels at T2 (peak immunoglobulin G) were correlated with TIR and TAR collected at the same time point, which refers to the glucose recorded during the 10 to 14 days before T2. No other correlations were found. Statistically significant correlations are highlighted in bold.

Abbreviations: TAR, time above range; TBR, time below range; TIR, time in range.

Females developed higher IgG than males (median IgG-AUC: 104.5 [98.9-118.0] vs 119.8 [109.1-130.7]; *P* = .03; median IgG OD at T2: 1.088 [1.036-1.187] vs 1.204 [1.145-1.365]; *P* = .057). Peak IgG was unrelated to age (*r* = 0.03; *P* = .88), BMI (*r* = −0.14; *P* = .53) or disease duration (*r* = 0.05; *P* = .84) and was similar between patients with and without chronic complications (1.18 [0.97-1.37] vs 1.11 [1.09-1.28]; *P* = .89). The IgG-AUC correlated with BMI (*r* = −0.53; *P* = .04) and disease duration (*r* = −0.53; *P* = .03), but not with age (*r* = 0.42; *P* = .098) or other parameters studied. Spike antibodies, neutralization titers, and glucose control/CGM profile were similar between pump users and multiple daily insulin injection (MDI)-treated patients. TIR tended to be higher in pump users compared to MDI-treated individuals (TIR at T0 68.71 ± 15.18% vs 58.83 ± 7.026%; *P* = .06), and CGM users tended to have better HbA_1c_ (7.0 ± 0.68% vs 7.5 ± 0.67%; *P* = .06) compared to those who do not wear a CGM, although the differences became weaker (*P* ≥ .35) when adjusting for multiple comparisons. However, after correction for multiple comparisons, CGM measures (TIR and TAR) were the only assessed variables showing statistically significant correlation with the IgG response to vaccine. Spike antibodies and neutralization titers were unrelated to antibody reactivity to 2 known T1D native autoantigens, insulin and GAD65 (*P* ≥ .39; data not shown).

## Discussion

Our data suggest that in T1D, despite the fact that HbA_1c_ does not influence humoral response to BNT162b2, a better CGM profile during the 2 weeks preceding vaccination is associated with higher antispike antibody binding and greater neutralization potency against SARS-CoV-2 after BNT162b2. Thus, achieving well-controlled glucose may have implications not only for prevention of diabetes complications but also for improving humoral immune response.

We did not find a significant association between glucose control assessed by HbA_1c_ and antibody response after vaccination. Consistent with our findings, 2 recent studies found that HbA_1c_ values in T1D and type 2 diabetes (T2D) were not correlated with antibody response following SARS-CoV-2 vaccination (11, 12). However, a larger study of T2D individuals highlights that 21 days after the first vaccine dose, neutralizing antibody titers and CD4 cytokine responses involving type 1 helper T cells were lower in T2D patients with HbA_1c_ levels greater than 7% than in individuals with HbA_1c_ levels less than or equal to 7%, evaluated at baseline before the first vaccine dose (13). The stronger correlation between IgG response and CGM, compared to HbA_1c_, may imply that the time frame immediately close to vaccination (as close as 2 weeks), is pivotal for an optimal immune response induced by vaccination. On the contrary, baseline HbA_1c_, which covers a longer time frame (eg, up to 3 months before vaccination), may not fully catch the effect of glucose on immune response following vaccine administration. Furthermore, HbA_1c_ does not take into account glucose variation, and may show a significant degree of discordance with CGM data in around 40% of T1D patients (14, 15).

To the best of our knowledge, our study is the first to evaluate the effect of glucose assessed by CGM during the 2 weeks preceding vaccine administration on the immunogenicity to a SARS-CoV-2 vaccine. Other groups have used CGM to assess the safety of SARS-CoV-2 vaccine on glucose control, finding mixed results. Heald et al (16) showed that vaccination against SARS-CoV2 can cause temporary changes in blood glucose levels in patients with diabetes, especially in those with lower HbA_1c_. By contrast, we did not find significant changes in TIR, TAR, or TBR following vaccination. Our data are closer to those by D’Onofrio et al (17), who observed no significant differences of TIR comparing the 3 days after vaccine administration with the 14 days preceding the vaccine. This finding is also consistent with a recent report showing no significant perturbation in CGM parameters after vaccine administration (18). Whether changes described by Heald et al (16) are a consequence of increased insulin resistance or transient impairment of β-cell function is unknown. Studies are ongoing to evaluate the safety of SARS-CoV-2 vaccine with regard to anti-islet autoimmunity in T1D, as well as to other autoimmune diseases (19). Interestingly, D’Addio et al (18) found that the majority of patients with T1D did not show any increase in the SARS-CoV-2–specific cytotoxic response compared with the robust increase observed in individuals without diabetes, despite an increase in anti-SARS-CoV-2 spike antibody titer. Our data highlight the importance of achieving optimal glucose control for sustaining the development of an effective humoral response in patients with T1D, who already suffer from impaired vaccine-specific cytotoxic activity. The lack of a measure of inflammatory markers, such as C-reactive protein, before and after the vaccination during follow-up did not allow us to investigate if inflammation might affect the TIR and consequently the antibody response. However, in our study, vaccination did not produce significant changes in antibody response to 2 known β-cell autoantigens (insulin and GAD65) or CGM profile, which may suggest that the relationship between spike antibody response and CGM profile is independent of inflammation.

Several mechanisms have linked hyperglycemia to reduced antibody responses, vaccination efficiency, and increased risk of infection to other pathogens, including impaired antigen recognition, altered cytokine expression, and immunosenescence (20, 21). Antigen glycation is another mechanism that we reported that impairs antigen-specific antibody response in T1D (5). Although a time-dependent decline in antibody levels might increase the risk of breakthrough infections, the antibody cutoff predicting such a risk is still unclear, which is a major study limitation. Our study was powered to detect large effect size in correlation analysis; therefore significant correlations of small and medium effect size might be missed, and the sample size represents therefore a limitation. Lack of a control group without diabetes is a study limitation. A strength of our approach is the use of multiple indices of glucose control, the use of a cell-based neutralization assay, and the length of follow-up. Larger studies that also include antigen-specific cell-based assays and assess different SARS-CoV-2 variants are needed to validate our findings and provide a more mechanistic insight into the relationship between glucose control and immune response to SARS-CoV-2.

In conclusion, our findings may suggest a relationship between glucose control assessed by CGM and antibody response after SARS-CoV-2 vaccination, highlighting the importance of achieving well-controlled blood glucose for COVID-19 prevention.

## Data Availability

Some or all data sets generated during and/or analyzed during the present study are not publicly available but are available from the corresponding author on reasonable request.

## Abbreviations

AUC: area under the curve
BMI: body mass index
CGM: continuous glucose monitoring
ELISA: enzyme-linked immunosorbent assay
HbA_1c_: glycated hemoglobin A_1c_
IgG: immunoglobulin G
MDI: multiple daily insulin injection
mRNA: messenger RNA
OD: optical density
T1D: type 1 diabetes
T2D: type 2 diabetes
TAR: glucose time above range
TBR: glucose time below range
TIR: glucose time in range
